# Effects of vessel traffic and underwater noise on the movement, behaviour and vocalisations of bottlenose dolphins in an urbanised estuary

**DOI:** 10.1038/s41598-017-13252-z

**Published:** 2017-10-18

**Authors:** Sarah A. Marley, Chandra P. Salgado Kent, Christine Erbe, Iain M. Parnum

**Affiliations:** Centre for Marine Science and Technology (CMST), Curtin University, Perth, Western Australia Australia

## Abstract

The potential disturbance of dolphins from tourism boats has been widely discussed in the literature, in terms of both physical vessel presence and associated underwater noise. However, less attention has been paid to the potential impact of non-tourism vessels, despite these being much more widespread and occurring in greater numbers throughout coastal dolphin habitats. The Indo-Pacific bottlenose dolphin (*T. aduncus*) community using the Fremantle Inner Harbour, Western Australia, is exposed to high levels of vessel traffic. To investigate whether behavioural responses could be occurring, a non-invasive combination of visual and acoustic monitoring was conducted using a theodolite and an autonomous acoustic logger. Dolphins significantly increased their average movement speeds in high vessel densities, but only for some activity states. Behavioural budgets also changed in the presence of vessels, with animals spending greater time travelling and less time resting or socialising. Finally, multiple whistle characteristics varied with rising levels of broadband noise, and other contextual variables. Despite being acoustically specialised for higher frequencies, dolphins had the strongest acoustic variation during low-frequency noise. This study highlights the complexity of disturbance responses in this species, confirming the need for consideration of both surface and acoustic behaviour alongside appropriate contextual data.

## Introduction

In coastal marine environments, vessel traffic is the most ubiquitous anthropogenic activity with the potential to disturb marine wildlife^1,2^. With wildlife-watching tours becoming increasingly popular, tourism vessels have been the focus of concerns regarding animal disturbance, particularly for coastal species such as bottlenose dolphins (*Tursiops* sp.) whose core habitats overlap with human activities and are relatively accessible for research programmes^3–17^. The literature reports varying degrees of tourism-related disturbance to dolphins – from relatively subtle changes in vocal behaviour to broad-scale animal movements away from the affected area. However, comparatively little attention has been paid to the potential impacts of recreational vessels, despite predictions of significant growth in vessel traffic over coming years^18^. Recreational vessels already occur in high numbers in some areas^19–23^, and thus have the potential to elicit dolphin behavioural responses despite not being actively engaged in wildlife-watching activities. This is again of particular concern with regard to coastal dolphin species, who are exposed to higher levels of human activity as well as the potential risk of cumulative impacts from repeated, long-term exposure to vessel traffic^24–26^.

Previous studies of vessel disturbance effects have often focused on dolphin displacement or changes in occurrence/site occupancy^4,6,27–29^. However, such an approach ignores the complex array of factors determining animal habitat-use in human-dominated seascapes^30^. Some studies have taken this next step and yielded evidence that dolphins may alter their movement patterns, for example by changing travel direction to orient away from/move around approaching vessels, beginning to travel erratically, or significantly increasing their travelling speeds^9,17,23,24,31,32^. Changes to activity states or behavioural budgets have also been reported, with travelling behaviour generally increasing with vessel presence, whilst resting and socialising become more infrequent^3,8,9,12,15^. Foraging is less predictable, with some studies reporting greater foraging activity in the vicinity of boats^11^, whilst others suggest the contrary^3,8,33^.

Less obvious to human observers are potential changes in dolphin acoustic behaviour in response to vessel traffic and associated underwater noise. Dolphins have been observed to adjust some characteristics of their whistles in elevated noise conditions or in the presence of tourism vessels, for instance by modifying their frequency range^5,13,34–37^, duration^5,13,35^, or the number of frequency modulations^36^. Increases in whistle production rates have also been reported^7,13,19^. These changes presumably stem from impaired communication efficiency as a result of vessel noise^38^. However, changes in acoustic behaviour can also occur in association with other factors, for example different surface behaviours, group sizes, group compositions, or even geographic location^5,13,39–45^. The plasticity of dolphin whistles requires consideration of context when investigating potential acoustic responses to human activities.

Yet, few studies control for additional explanatory variables to provide suitable context, or recognize that behavioural and acoustical responses are not necessarily mutually exclusive. In practice, it can be difficult to disentangle the effects of physical boat presence and associated noise^28,46^, with results also being confounded by the fact that behavioural studies are frequently conducted from research vessels that could themselves elicit responses^12,13,34^. Moreover, the way in which one population responds to vessel traffic does not necessarily mean that all populations will respond in the same manner^46^. Dolphin communities vary in terms of their vessel exposure histories, home range environmental conditions, and survival pressures such as prey availability or predator densities. Thus, dolphin communities should be examined on a “case by case” basis, especially when considering the conservation of small communities or populations particularly susceptible to threats due to their small numbers and low reproductive rates^25,26^. There is a need for studies investigating a range of dolphin-vessel contexts with regard to recreational vessel traffic, from both behavioural and acoustical perspectives, preferably conducted from land-based research platforms.

**Figure 1:**
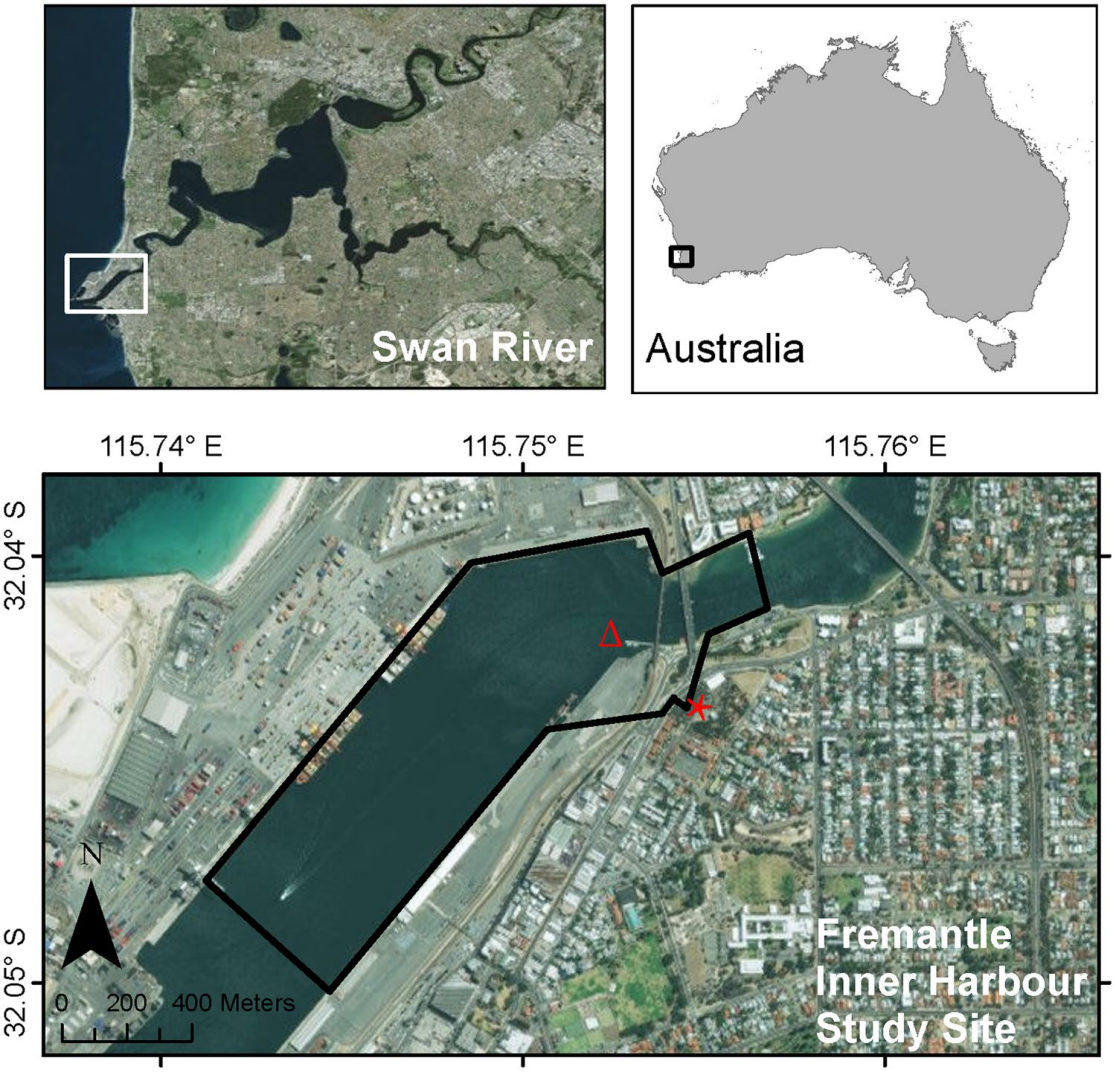
Map of the Fremantle Inner Harbour study area, showing the location of the theodolite hill-top station (red star) and acoustic logger deployment (Δ). Maps were created in ArcGIS® (version 10.1) by ESRI (www.esri.com) using the World Imagery basemap (sources: Esri, DigitalGlobe, Earthstar Geographics, CNES/Airbus DS, GeoEye, USDA FSA, USGS, Getmapping, Aerogrid, IGN, IGP, and the GIS User Community; http://goto.arcgisonline.com/maps/World_Imagery).

Here, we consider the behavioural and acoustical responses of a dolphin community in Western Australia to vessel traffic and underwater noise. Recreational vessel traffic is on the rise in Australia, with the country currently experiencing rapidly growing recreation use of marine resources. Between 1999 and 2009, there was a 44% increase in the number of recreational vessels registered in Western Australia^47^. There are currently approximately 95,000 recreational vessels registered in the state, with over half of these within the state capital of Perth (J. Nunn; personal communication, 21st January 2014). Consequently, the Swan-Canning River system, which flows through the centre of Perth, receives high levels of vessel traffic^22^. The Fremantle Inner Harbour (Fig. 1) is part of an industrial port at the river mouth, and acts as a gateway between the Swan River and the Indian Ocean. It has been identified as the anthropogenically-noisiest site in the river, with transits of up to 56 vessels per hour^22,48^. But despite being a busy, noisy site, the Fremantle Inner Harbour is frequently used by the river’s resident community of Indo-Pacific bottlenose dolphins (*T. aduncus*). This community consists of approximately 18 adults, plus several juveniles and calves, which use the harbour as one of several “hotspots”^49–52^. Dolphins have been shown to remain within the Fremantle Inner Harbour during periods of high vessel traffic, with no alterations to their presence/absence, number of sightings, or duration of time spent in this area in relation to vessel density^22^. However, behavioural responses could be occurring at a finer scale, despite the lack of site avoidance. Since the manner in which dolphins respond to vessel traffic could have implications to their health, the effects of vessel traffic and underwater noise must be quantified.

This study aims to investigate the behavioural responses of bottlenose dolphins to vessel traffic and acoustical behaviour in varying underwater noise conditions in the Fremantle Inner Harbour. To achieve this, dolphin movement speeds, activity states and whistle characteristics in varying levels of vessel traffic and underwater noise were examined. Data were collected using autonomous acoustic recordings and land-based visual observations to minimise bias from the presence of a research vessel. Additionally, contextual covariates (such as behaviour, group size, and calf presence) were recorded for inclusion in analyses. The resulting knowledge on fine-scale behavioural response to vessels will improve management practices and conservation outcomes.

## Results

A total of 217 h of visual observations were undertaken during 83 surveys over three years. Surveys ranged in duration from 0.5 to 4.1 h, and yielded a total of 185 dolphin group sightings in the study area. Of the cumulative 83 h of 5-min samples collected, 28% of dolphin observations occurred in high vessel-density conditions (i.e. a median of over three vessels per 5-min sample).

### Dolphin Movement Speed

Average dolphin group speeds ranged from 0.03 to 2.67 m/s per 5-min sample (n = 782), and differed significantly among the five activity states, as defined in Supplementary Table S1 (Kruskal-Wallis Test; *X* ^2^ = 77.381, df = 4, p < 0.001).

**Figure 2:**
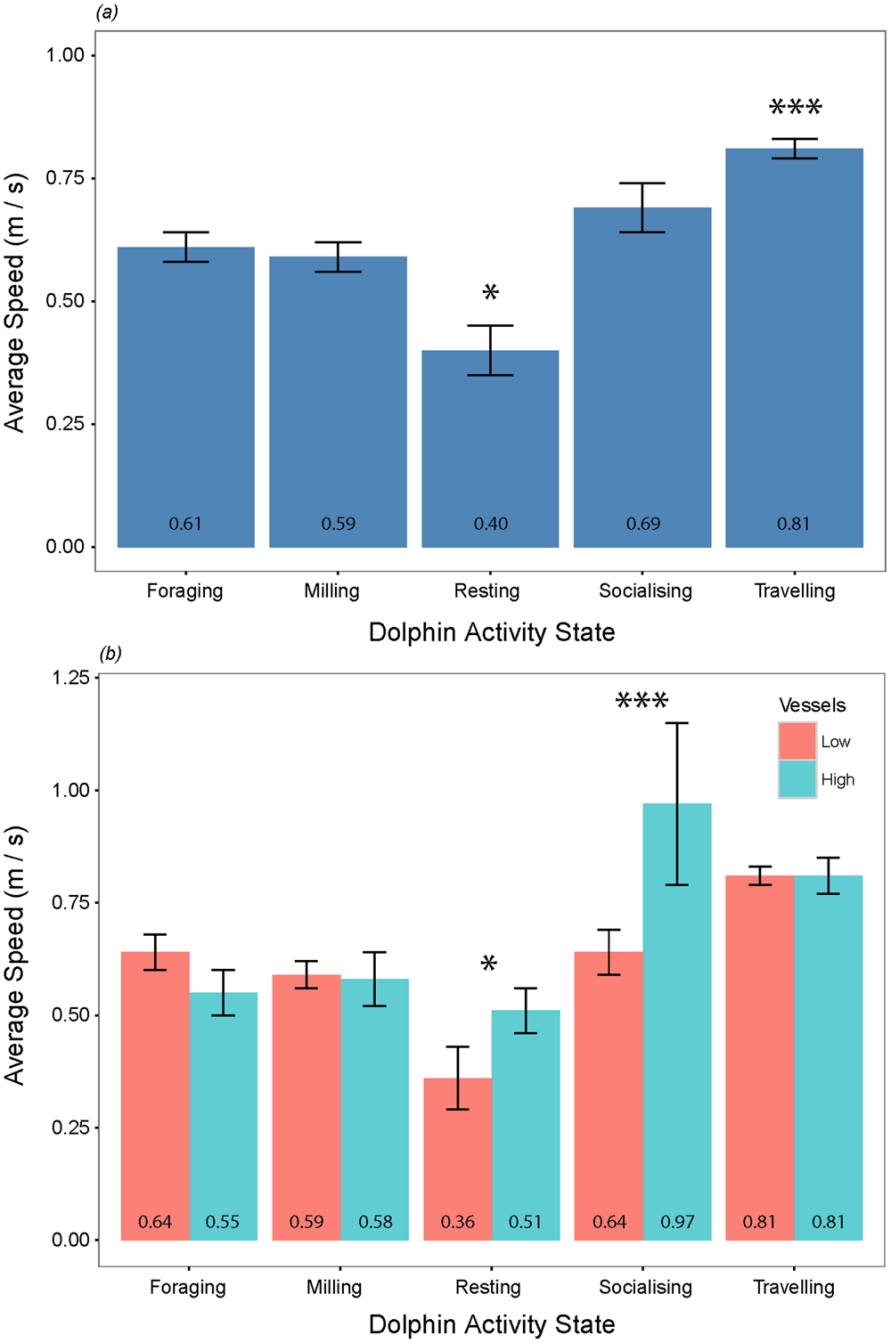
Average dolphin group movement speed according to (**a**) activity state, and (**b**) high and low vessel densities for each activity state. Definitions for each activity state are provided in Supplementary Table S1. Numbers represent the average speed in m/s. Error bars represent the standard error. Significance level: ***≤0.001; **≤0.01; *≤0.05.

Average speeds for travelling were significantly higher than during all other activity states (Mann-Whitney U Tests; p < 0.01), whilst averaging speeds during resting were significantly lower than during all other activity states (p < 0.05; Fig. 2a). There were no significant differences (p > 0.05) between average speed during foraging, milling and socialising.

Activity states were considered independently for different levels of vessel traffic (low or high). Mann-Whitney U Tests showed no significant differences (p > 0.05) in average speed between vessel contexts for travelling, foraging or milling. Both resting and socialising average speeds significantly increased with increasing vessel densities (respectively, U = 176.5, p = 0.01673; U = 503, p = 0.04216; Fig. 2a). However, these results should be interpreted with caution given the low sample sizes associated with high vessel densities (high n = 10 and 11, respectively).

### Dolphin Activity State

A total of 714 activity state transitions were extracted from sequences of two adjacent 5-min samples. Transitions included 462 in Low vessel traffic, 139 in High vessel traffic, 56 in Increasing vessel traffic, and 57 transitions in Decreasing traffic conditions.

**Figure 3:**
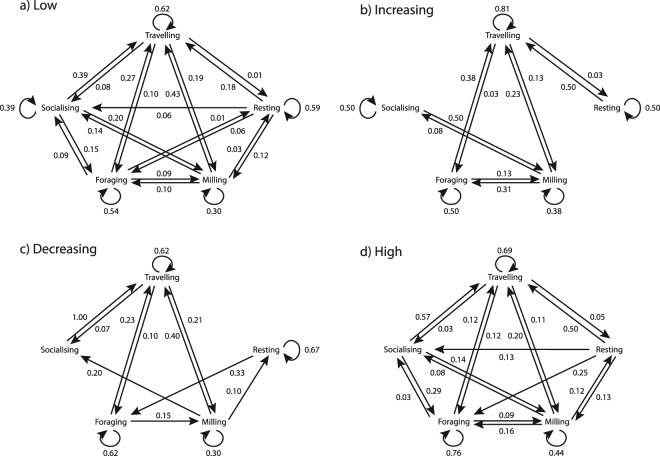
Markov chains representing transition probabilities between preceding and succeeding activity states in four different vessel contexts: (**a**) Low; (**b**) Increasing; (**c**) Decreasing; and (**d**) High vessel traffic. Definitions for each activity state are provided in Supplementary Table S1.

In any vessel traffic context, transition probabilities indicated that dolphins were most likely to continue travelling or foraging if that was the behaviour they were engaged in previously (with some exceptions, Fig. 3). At High vessel densities, resting was most likely to transition to travelling, as was socialising. For Decreasing vessel densities, milling and resting animals were both most likely to begin travelling. By contrast, at Increasing vessel densities, resting and socialising were as likely to continue as they were to transition into travelling or milling activities.

Small sample sizes for some behaviours and vessel contexts prevented these from being statistically tested. Out of 75 possible activity state comparisons between the Low vessel density and the other vessel impact conditions, 12 statistical comparisons were possible. For Low versus Increasing, activity state comparisons were made between travelling-to-travelling and milling-to-milling transition probabilities. For Low versus High, activity state comparisons were travelling-travelling, travelling-milling, travelling-foraging, milling-travelling, milling-milling, milling-foraging, and foraging-foraging. Finally, for Low versus Decreasing, activity state comparisons were travelling-travelling, travelling-milling and foraging-foraging. Of these 12 possible comparisons, only three were significantly different from the Low vessel context. When vessel density was Increasing, the probability of travelling-to-travelling (i.e. no transition in travelling behaviour) increased (z = 2.1, p = 0.038). When vessel density was High, the probability of milling transitioning to travelling decreased (z = 2.1, p = 0.036) and the probability of foraging to continue (foraging-to-foraging transition) increased (z = 2.2, p = 0.028).

**Figure 4:**
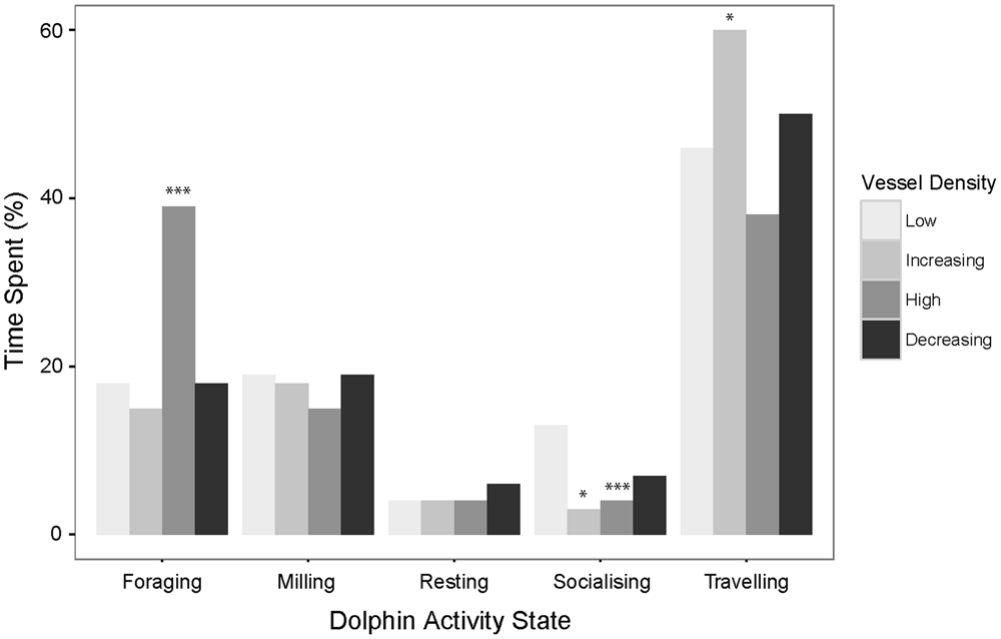
Behavioural budgets of dolphins in four different vessel contexts (Low, Decreasing, Increasing and High). Results of Z-Test comparisons between Low and other vessel contexts are indicated: ***≤0.001; **≤0.01; *≤0.05.

Behavioural budget comparisons indicated that dolphins spent a greater proportion of time travelling (z = 2, p = 0.0476) and less time socialising (z = 2.2, p = 0.0292) when exposed to Increasing vessel densities than in Low conditions (Fig. 4). When vessel density was High, the proportion of time spent socialising was also significantly reduced (z = 3, p = 0.0029), whereas foraging significantly increased relative to Low vessel densities (z = 5.2, p < 0.001). The proportion of time spent in different activity states was not significantly different between Low and Decreasing vessel densities (all p > 0.05).

### Whistle Characteristics

All of the whistle characteristics measured were associated with mean broadband noise level (NL_BB) and dolphin activity state (Supplementary Table S2), and to a lesser extent with group size and the presence of calves. GAMs were fitted to 161 of the 164 whistles, after three were removed as outliers. GAMs included all whistle characteristic measures as response variables, except for the number of breaks or saddles due to small sample sizes (<20 whistles).

**Figure 5:**
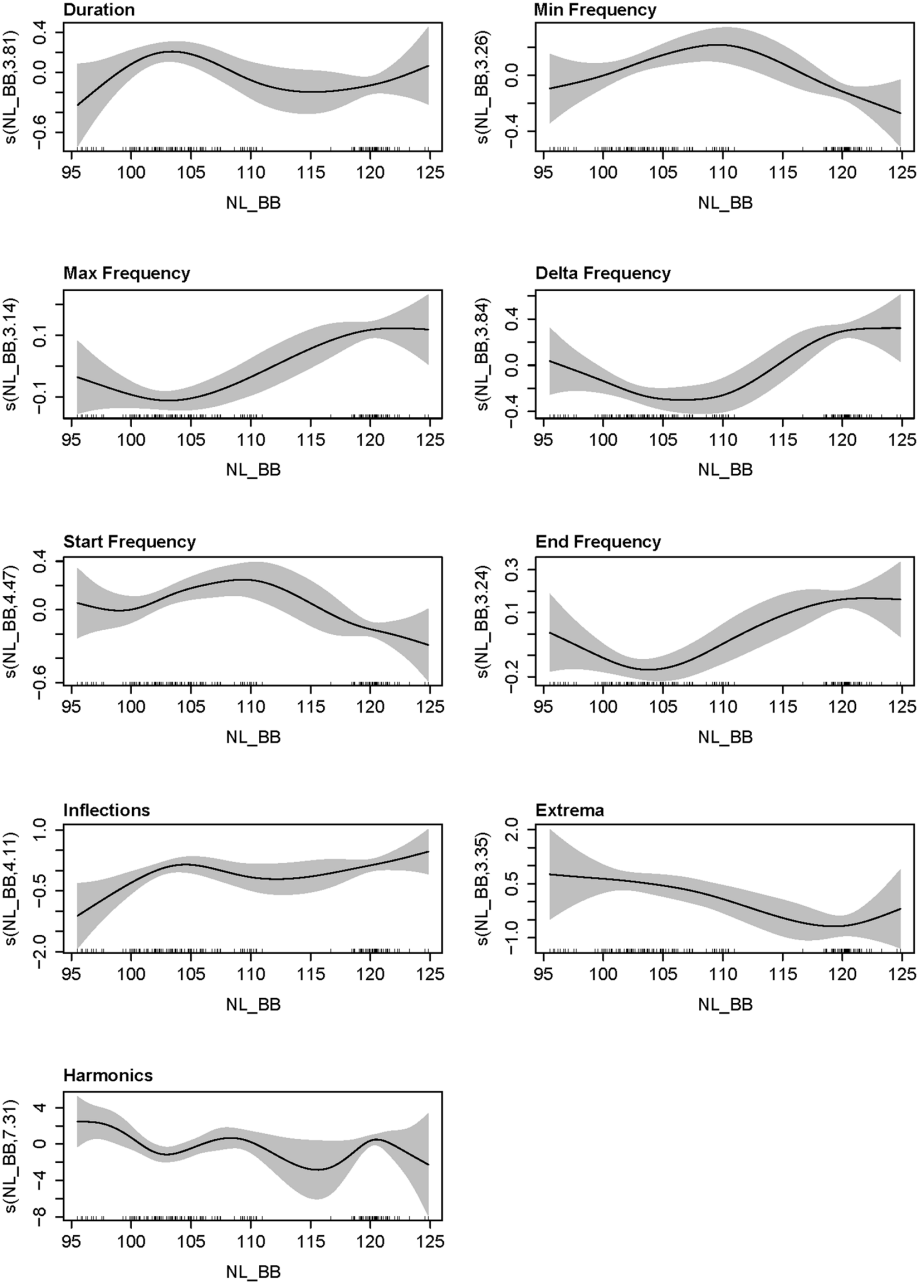
Results of the nine whistle characteristic GAMs which selected broadband noise level (NL_BB) as a significant explanatory variable.

NL_BB over the whole recording period were approximately 110 dB re 1 µPa rms (10 Hz–48 kHz). Whistle duration, minimum frequency, start frequency, number of extrema and presence of harmonics decreased with increasing NL_BB (Fig. 5). In comparison, maximum frequency, end frequency, and change in frequency (delta frequency) typically increased with increasing noise.

**Figure 6:**
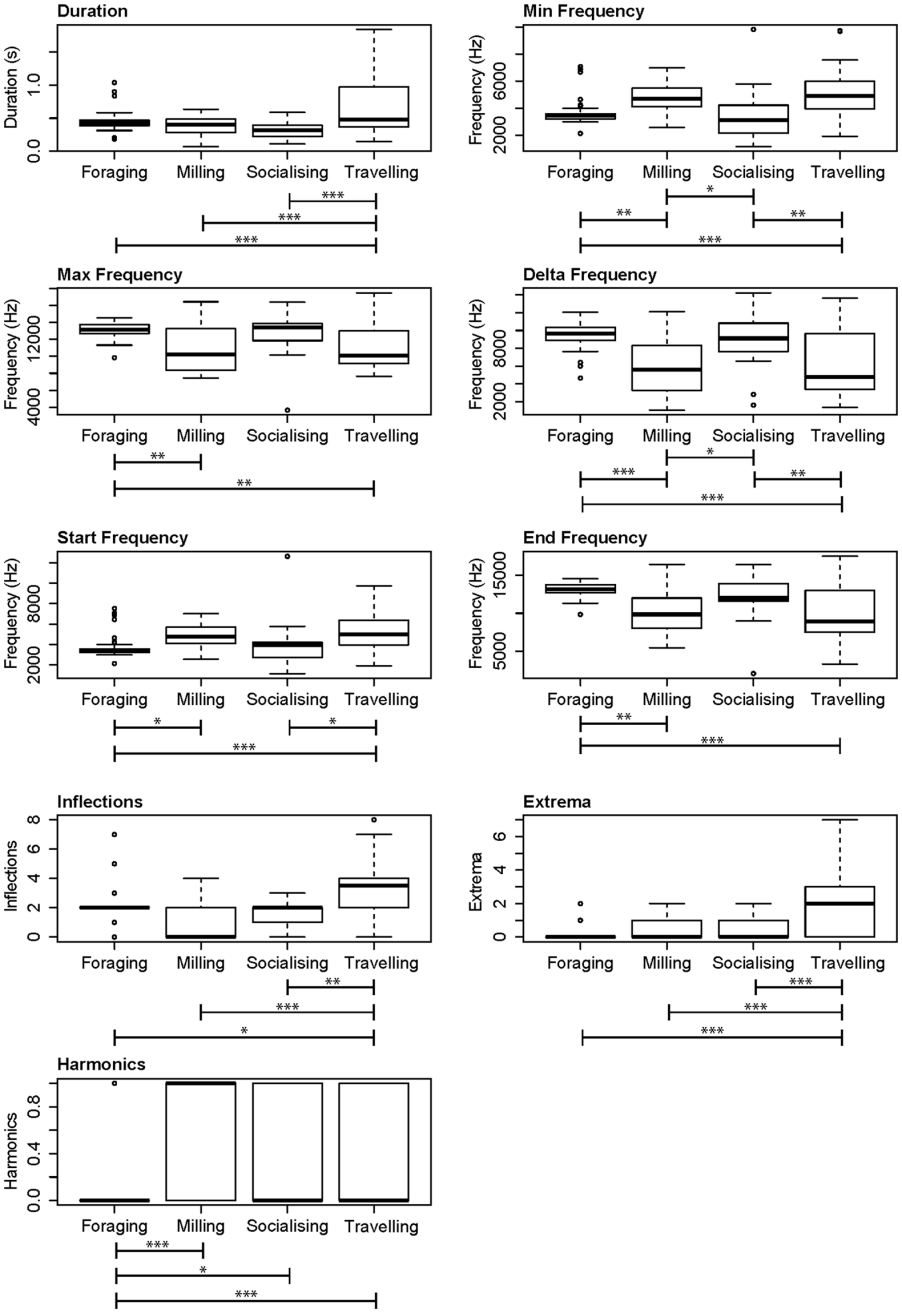
Results of the nine whistle characteristic GAMs which selected dolphin activity state as a significant explanatory variable. Post-hoc tests were used to test for differences between behavioural states. Significance level: ***≤0.001; **≤0.01; *≤0.05.

The majority of the whistles were produced when the predominant activity state of the group was travelling (51%), followed by foraging (25%), milling (13%) and socialising (10%). No high-quality whistles were obtained whilst animals were resting. Differences in all whistle characteristics occurred between travelling and foraging dolphins (Supplementary Table S2; Tukey post-hoc tests, Fig. 6). All frequency measurements and harmonics were significantly different between milling and foraging dolphins, representing two-thirds of all whistle characteristics. Similarly, two-thirds of whistle characteristics significantly differed between travelling and socialising dolphins, namely: duration, minimum frequency, delta frequency, start frequency, inflection points, and extrema. Fewer differences existed between the remaining activity state pairs. Differences included those in duration, inflection points, and extrema between travelling and milling; minimum and delta frequencies between socialising and milling; and the presence of harmonics between socialising and foraging.

**Figure 7:**
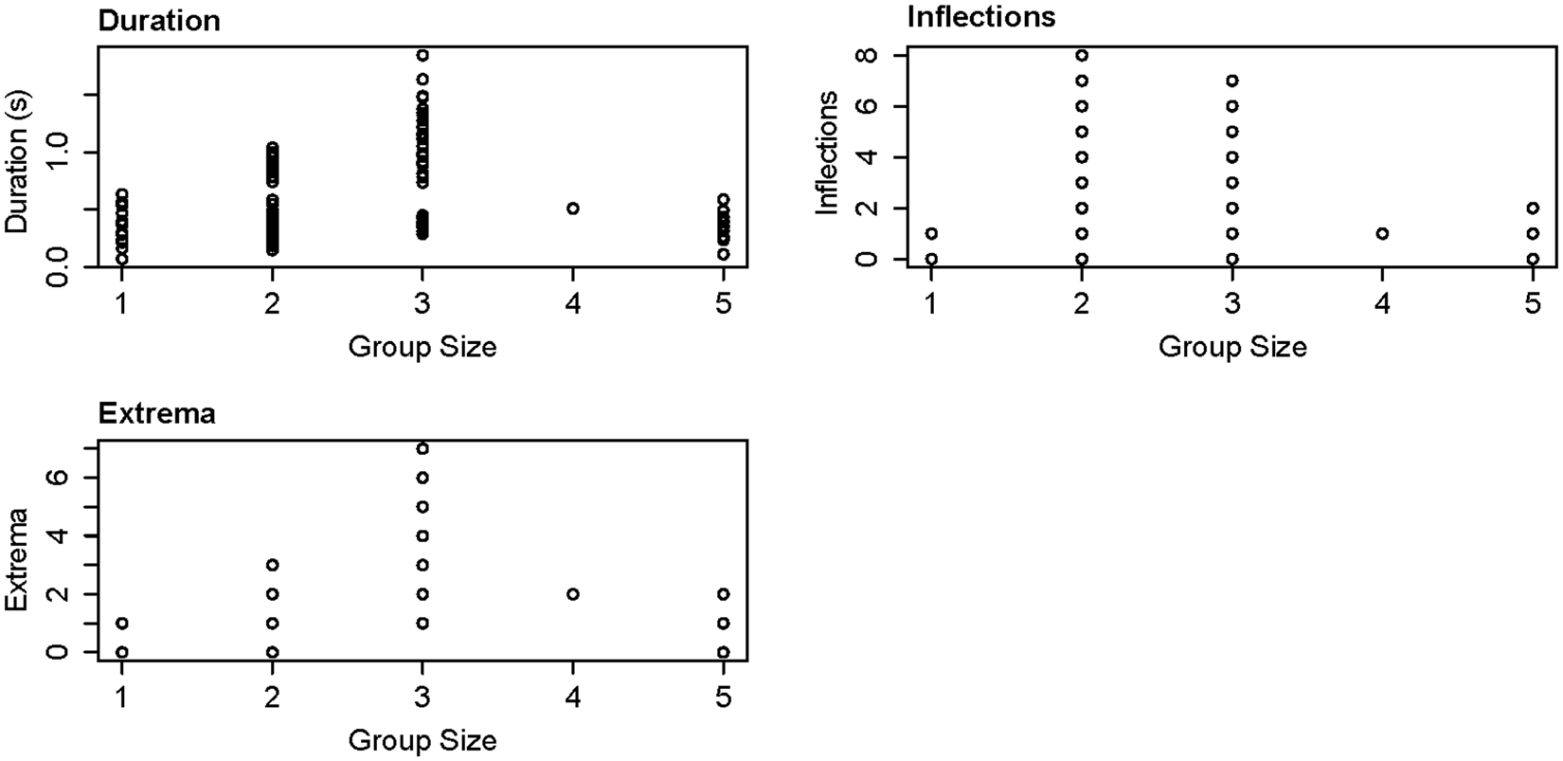
Results of the three whistle characteristic GAMs which selected dolphin group size as a significant explanatory variable.

Whistles were all produced in the presence of single groups composed of 1 to 5 dolphins (mean = 2.4). Group size was only significantly related to whistle duration, maximum frequency, delta frequency, inflection points and extrema (Supplementary Table S2; Fig. 7). Values for these characteristics were typically highest for group sizes of two or three animals.

**Figure 8:**
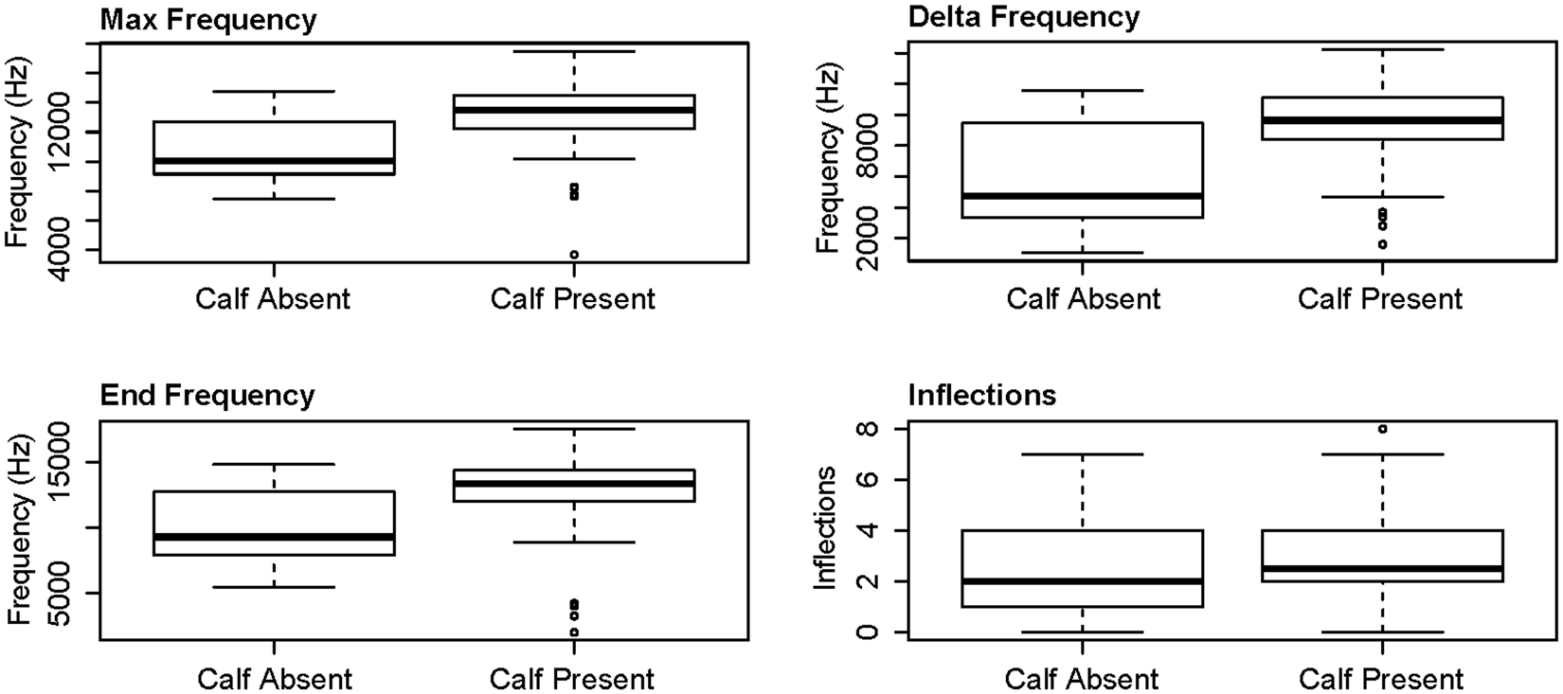
Results of the four whistle characteristic GAMs which selected the presence of dolphin calves as a significant explanatory variable.

Of the whistles analysed, 41% were produced in the presence of dolphin calves. The presence of a calf significantly affected the maximum, delta and end frequency characteristics and inflection points (Supplementary Table S2; Fig. 8), with all mean values increasing.

Investigation into noise levels at specific frequency levels revealed associations between whistle characteristics and twelve octave-band levels (OBLs). However, the bands showed high collinearity, and only three (with VIFs < 5) were accordingly included in GAMs: 1 kHz, 16 kHz and 32 kHz. While dolphins have been reported to show sensitivity to noise at 100 Hz^53,54^ and even to produce sounds with fundamental frequencies as low as 260 Hz^55^, only the three OBLs listed above were included as they are within the frequency range of most dolphin whistles recorded. The 1 kHz OBL (NL_1000) was associated with all whistle characteristics in the best fitting models (Supplementary Table S3, Supplementary Figure S4). Whistle duration, minimum frequency, start frequency, extrema and harmonics generally declined when NL_1000 was high, whereas the remaining frequency characteristics typically increased. Inflections showed variable results. The 16 kHz OBL (NL_16000) was associated with maximum frequency, inflections and harmonics, which all decreased as NL_16000 increased (Supplementary Table S3, Supplementary Figure S5). Finally, the 32 kHz OBL (NL_32000) was associated with six whistle characteristics; typically with increasing duration, extrema and harmonics, decreasing delta frequency and end frequency, and displaying mixed results for inflections (Supplementary Tables S3 and S6). Based on the number of characteristics affected, lower-frequency noise generally was overall more strongly associated with dolphin whistle characteristics than higher-frequency noise.

## Discussion

This study showed significant changes in bottlenose dolphin behaviour in Fremantle Inner Harbour in association with high vessel densities and underwater noise. Animals’ responses to stressors can take a variety of forms. For instance, whilst a previous study conducted in Fremantle Inner Harbour documented reduced dolphin detection rates during pile-driving activity^56^, more recent research at the same site found no alteration in the occurrence of dolphins in heavy vessel traffic^22^. Here, dolphins did appear to change movement speeds and activity states in response to vessel density, whilst whistle characteristics differed in varying underwater noise conditions.

While the average travel rates of dolphin groups were a function of activity state, their movement speed was also affected by vessel traffic. Animals moved fastest during travelling and slowest during resting activities. Resting and socialising groups significantly increased their average speed when vessel density was high. Captive studies suggest that higher swim speeds induce greater metabolic rates^57,58^. The risk of impact may be particularly high for resting animals, in that they expend more energy than they would otherwise and their quality of rest is reduced. Resting is important for overall animal health. Disruption to resting activities can induce stress as well as reduce energy reserves, which ultimately affect an animal’s condition and immune function^12,59–62^. Thus an increase in dolphin movement speed whilst resting could have both physiological and energetic consequences at the individual-level.

Resting and socialising activity states were mainly observed during low vessel densities. While there was an observed increase in resting after high vessel densities, the increase was not statistically significant. This may ensue from the limited power of the analysis caused by small sample sizes for this behaviour. Resting occurring during low vessel densities could reflect animals that were capitalising on less-busy periods to rest following exposure to high vessel traffic. In addition, female dolphins mainly nurse their calves while resting^9^. The slight increase in resting behaviour after exposure to high vessel densities could therefore represent an opportunity for nursing. There were no differences between behavioural budgets during low and decreasing vessel contexts for any of the activity states. Thus, dolphins may make use of low or decreasing vessel density periods to return to their original activities.

Time spent socialising significantly shortened during increasing and high vessel densities. Socialising behaviour is important for the maintenance of conspecific relationships, calf play, and mating opportunities. If these behaviours are repeatedly interrupted there could be long-term, population-level consequences. For example, fewer successful mating attempts could result in lower reproduction rates and thus influence population dynamics. Play behaviour has been shown to be important to the development of dolphin calves as a method of learning physical manoeuvres, object manipulation, problem-solving skills, social behaviours, and foraging methods^63–65^. But play behaviour is less likely to occur in stressful situations^66^, and could also be disrupted if socialising events become less frequent. Decreased socialising opportunities for calves may therefore impact reproductive or foraging success when they become adults.

Dolphins spent more time travelling whilst vessel density was increasing. Travelling typically occurs at greater average movement speeds than other activity states, so may lead to increased energetic demands. Time spent travelling also leaves fewer opportunities to engage in other vital behaviours such as foraging or resting. Once vessel density was high, dolphins increased the time they spent foraging. Christiansen *et al*.^11^ also reported an increase in foraging when bottlenose dolphins encountered tour boats; yet many other studies have provided evidence to the contrary^3,8,33^. Increased foraging activity could be an attempt to compensate for energy lost during disturbance, or could alternatively reflect changes in prey behaviours. For example, fish may scatter in response to vessel transits, making them easier to capture. The observed foraging behaviour may also have been erroneously reported. What was perceived as foraging behaviour could have been an increase in transitions to diving in the presence of tour boats, which has previously been hypothesised to represent vessel avoidance by utilising a different part of the water column^15^. Because high vessel densities characterised approximately 28% of the dolphin behavioural samples collected, dolphins could be expected to spend a significant proportion of time expending energy by altering their activities. If these indeed result in increased energy requirements, reduced fitness at both individual and population levels may occur^17^.

Different vocal behaviours were also observed in contrasting noise conditions associated with presence of vessel traffic (among other sources). When noise levels were relatively high (≥110 dB re 1 µPa rms [10 Hz–48 kHz]), whistles were typically short with a wide frequency range and had multiple inflection points and fewer extrema (similar to ‘complex’ whistles). At higher noise levels, harmonics were more frequently absent, though this could be the result of masking by noise at overlapping frequencies. Noise at lower frequencies was associated with the broadest differences in whistles, as differences were observed in all characteristics.

Whilst whistle characteristics varied with noise levels, they also varied according to activity state, group size and calf presence. The whistles of foraging and socialising groups were typically associated with whistles of short duration and wide frequency range, starting low and ending high, with few inflections and extrema (similar to ‘upsweeps’). Whistles recorded in the presence of milling dolphins were also relatively short but had a smaller frequency range, with low numbers of inflection points and extrema, though they generally included harmonics. Travelling groups exhibited longer-duration whistles that spanned a narrower frequency range but contained the highest numbers of inflections and extrema (similar to ‘sine’ whistles). Whistles recorded when dolphin duos or trios were present were typically of longer duration, with higher maximum and delta frequency values (but unchanged minimum frequency) and contained a greater number of inflections and extrema compared to singletons and group of four and five animals. And finally, whistles recorded when calves were present typically started at a lower frequency and ended at a higher frequency (giving a correspondingly high delta-frequency), plus had lower minimum and higher maximum frequency values and a greater number of inflection points.

Although the whistle sample size was relatively large, the number of unique dolphin groups from which the samples were derived was very limited (n = 11). Merely three of these groups produced the majority of whistles analysed in this study, with remaining groups contributing < 20 whistles each. These three groups were engaged in one of two predominant activities (foraging or socialising), and were of specific group sizes (2 or 3 individuals) and compositions (two groups without calves, one with a calf). In this context, and despite the lack of apparent collinearity between noise levels and activity state, group size or presence of calves, it remains unclear whether noise is ultimately a proximate driver of the observed variation in whistle characteristics. Future research should focus on disentangling the effects of group from activity state, group size and calf presence. Furthermore, it was not possible to identify which individuals were producing the whistles. It is therefore possible that some individuals were re-sampled in this study, and that signature whistles unique to individual dolphins were captured multiple times. Bottlenose dolphins’ whistles are highly plastic, which facilitates adaptation to dynamic environmental conditions^35^ and communication requirements. In the future, it would be beneficial to examine group composition in terms of which individual dolphins are present with regards to alterations in whistle characteristics in association with noise levels. This will help to clarify the most important drivers of whistle variations.

Other recommended future work involves quantifying energetic consequences of disturbance, collecting finer-scale information on behavioural and acoustical responses, comparing dolphin responses within the Fremantle Inner Harbour with those of other Swan River sites, and determining which vessel characteristics may elicit responses. Previous studies have found that dolphin metabolic rates increase during periods of sound production, and may take several minutes to return to resting values^67^. Similarly, if increased vessel density causes increases in travelling or foraging behaviours that are associated with greater energy demands for communication, the effects from boat traffic could be potentially magnified. This not only carries implications for dolphin energetic requirements but also highlights the complexity of disturbance responses in this species. Quantifying the impacts of vessel traffic on dolphin metabolism from a conservation physiology perspective would also be further enhanced by obtaining behavioural response information at an even finer resolution, e.g. by monitoring surfacing patterns, dive times, inter-animal spacing, within-site movements, whistle rates and individual behavioural events^7,9,13,16,17,19,24,68,69^. How the responses of Swan River dolphins vary between sites, as well as in comparison with other dolphin communities, could highlight areas of conservation priority. For example, some locations may be mainly used for travelling or foraging, whereas others may act as resting or nursing grounds with an inherently higher risk of disruption. Individuals may even select to use ‘quieter’ sites for resting or socialising to compensate for decreases in these activity states at busier or noisier sites. Some populations may naturally be less energetically-stressed than others, thus being able to better cope with greater energy demands during disturbance. Sites also vary with regard to their use by vessels. Finally, considering the range of vessel types observed within the Fremantle Inner Harbour^22^, it would be beneficial to investigate dolphin responses in terms of vessel manoeuverability, speed and noise characteristics. This could inform guidelines for boat operations within important dolphin habitats, and would be particularly relevant should dolphin-watch tourism activities (which are not currently present in the Swan River) commence. Tourism operations could result in behavioural responses occurring more frequently and intensely, and represent a broader range of responses than those observed in this study. Overall, this study expands upon previous behavioural response work on this dolphin community^22,^ ^56^, and sets the scene for future research.

In conclusion, dolphins within Fremantle Inner Harbour changed their movement speeds and activity states in increasing vessel traffic. Whistle characteristics varied with different underwater noise conditions, although whether these were changes in response to noise remains unclear. While dolphins’ behaviour changed, they did not avoid the site altogether in response to high vessel densities^22^. The behavioural responses observed in this study raises concerns regarding the potential for increased energetic demands for dolphins, through increased movement speeds, greater time spent travelling, less time resting/socialising, and increased vocal effort. However, further work is needed to determine whether some of these energy requirements could be offset by the energy gained foraging at this site. The capture probability of prey responding to transiting vessels may even be improved. Further research is required to examine dolphin target prey species, prey responses to vessel traffic, and the possibility of vertical avoidance strategies by dolphins within the Fremantle Inner Harbour. The complexity of disturbance responses in this species justifies the inclusion of contextual information in future behavioural response studies.

## Methods

### Data Collection

Land-based visual surveys were conducted from a 32 m vantage point (32.0436°S, 115.7542° E) overlooking the Fremantle Inner Harbour (Fig. 1). Surveys occurred in May – June 2012, February – June 2014 and April – June 2015, covering months which coincided with a period of peak dolphin sightings in this area^52^. Surveys were only undertaken in good weather conditions (low glare, high visibility, Beaufort <4, temperatures <35 °C).

A surveyor’s theodolite (TopCon GTS-603 AF Electronic Total Station) was used to obtain positions of dolphin groups and vessels, with data recorded in real-time using the Vadar (vr 2.00.01b) software (E. Kniest, University of Newcastle). When dolphins were sighted, an initial theodolite position was taken, whilst group size, composition and activity state were noted by observers using 7 × 50 mm Bushnell binoculars. A “group” was defined as an association of dolphins in close proximity engaged in the same direction of travel and/or general activity. Five activity states were used: foraging, milling, resting, socialising, and travelling. These activity states were mutually exclusive, and similar to those used in other studies^15,70^. The predominant activity state was considered as the activity based on the most frequently observed behaviour. A theodolite position was taken at every surfacing interval while within the study area, with the exception of times when the surfacing interval was too brief to obtain an accurate reading. Vessels were tracked continuously during their time in the study area (irrespective of dolphin presence), using the same theodolite. Vessel identity was noted based on either the name or a relevant physical description (hull/canopy colour, crew, distinguishing features, etc.) to allow continual tracking, regardless of the number of vessels present.

Each survey was divided into 5-min samples, starting at integer multiples of 5 min. For each 5-min behavioural sample, the dolphins’ predominant activity state, group size and composition were noted. To determine the context of vessel traffic, vessel densities were calculated based on the number of vessels present in the study area during any 5-min sample. Vessel densities were then categorised according to a cut-off value based on the median number of vessels observed in any 5-min sample across the whole study period (median = 3). This resulted in ‘low’ and ‘high’ vessel traffic respectively being 0–3 and 4–16 vessels per 5-min sample.

Acoustic data collection took place concurrently with the April – June 2015 visual surveys. A high-frequency autonomous acoustic logger, equipped with a programmable 16-bit digital sound recorder (made by Wildlife Acoustics Inc) and an external hydrophone (Reson TC4033-1, sensitivity −202.8 dB re 1 µPa/V), was deployed within the Fremantle Inner Harbour (32.0420 S, 115.7528°E; Fig. 1). The hydrophone entered the housing via a bulkhead connector to an impedance matching pre-amplifier with 20 dB gain. Digitised recordings were written to four 128 GB SD cards. Before deployment, the logger was calibrated by applying white noise of known power spectral density. An 8 Hz high-pass filter was employed to filter out high levels of low-frequency noise, thus enhancing the dynamic range of the recorder at the frequencies of interest. The logger recorded at a duty cycle of 10 min every 15 min at a sampling frequency of 96 kHz.

### Movement Speeds

Vadar automatically calculates a target’s speed of travel based on the time and location of the previous versus the current theodolite position. Only samples with a minimum of two theodolite measurements were used, to ensure sufficient observations for speed calculations. This also ensured reliable speed estimates for animals ‘looping’ around the area. The average speed across all theodolite positions was then calculated for each 5-min sample, and paired with the corresponding dolphin activity state and level of vessel traffic, as defined above.

Analyses were undertaken by using a Kruskal-Wallis Test to determine whether activity state was associated with average speed. Subsequent Mann-Whitney U Tests were used to identify the source of differences. A series of Mann-Whitney U Tests were then conducted comparing average speeds during low and high vessel traffic for each activity state. These non-parametric tests were applied because a Shapiro-Wilk Test showed that the data were not normally distributed. All tests were conducted in R using the *stats* package^71^.

### Activity States

Assessing variation in animal behaviour is difficult due to the inherent temporal dynamics of activity states and changing contextual conditions. To quantify this temporal dependence and how context may change behaviour, Markov chains were employed to model dolphin responses to vessel density based on surface behaviour. First-order Markov chains were chosen, as these describe events which depend on the immediately preceding event^72^; thus, each activity state was examined with regard to the immediately preceding behaviour. The following methodology is an adaptation of Lusseau^15^, where the background statistics are extensively described.

Vessel traffic conditions were categorised as either: (1) Low, (2) High, (3) Increasing, or (4) Decreasing. These definitions considered the preceding and succeeding samples, and were based on the cut-off value of 3 vessels per 5-min sample as described above. For example, if both the preceding and succeeding behaviours occurred at low vessel densities, conditions were classed as ‘Low’. However, if the preceding and succeeding behaviours occurred at low and high vessel densities respectively, conditions were classed as ‘Increasing’.

A “transition” occurred when a dolphin group changed from a preceding activity state *i* to a succeeding activity state *j*, under a given vessel context. These transitions were recorded in a three-way contingency table, considering preceding versus succeeding activity state for each vessel traffic condition. Based on this table, transition probabilities were computed to detect the effect of changing vessel densities on dolphin activity states. The transition probability was calculated by dividing the number of transitions by the total number of times *i* was seen as the preceding behaviour:

$${p}_{ij}=\,\frac{{a}_{ij}}{{\sum }_{j=1}^{5}{a}_{ij}},\,\sum _{j=1}^{5}{p}_{ij}=1,$$

where *a*_*ij*_ is the number of transitions observed from *i* to *j*, and *p*_*ij*_ is the transition probability from *i* to *j*. The transition probabilities from the Low chain were then compared to those of the three other vessel traffic conditions using a Z-Test for proportions via the online EpiTools calculator (http://epitools.ausvet.com.au) where cell sample size was greater than five.

The activity budgets of dolphins in each vessel traffic condition were computed based on these same transition probabilities. These were arranged in four matrices, which were used to calculate the left eigenvectors of the dominant eigenvalues of each matrix. Calculations were performed in Matlab (Version 2014a, The Mathworks Inc.) based on code provided in Caswell^72^. Behavioural budgets for Low vessel contexts were compared with the other three contexts via a Z-test for proportions.

### Whistle Characteristics

Concurrent visual and acoustic records during May/June 2015 totalled approximately 35 h, during which 336 dolphin whistles were recorded. Marley *et al*.^73^ describes how dolphin whistles were identified and characterised based on these recordings. The current study uses 164 of these whistles, which were deemed to be ‘high quality’^73^, to investigate the relationship between underwater noise and dolphin whistles.

Separate Generalised Additive Models (GAMs) with contextual covariates were fitted for each of the 11 whistle characteristics described in Marley *et al*.^73^. The explanatory variables initially considered for inclusion in each model were: activity state, group size, calf presence, and broadband noise levels. To attribute these data, whistles were first paired with a visual observation record of the dolphins, based on the temporally closest theodolite data. If there was no theodolite observation within 10 min, the whistle was excluded from analysis. Secondly, for each whistle the broadband noise levels (NL_BB) were calculated as the root-mean-square sound pressure level over the 2 s period immediately prior to each whistle. There is no literature regarding the time delay required before increased noise levels induce an acoustic change in dolphin whistles. However, studies of the Lombard effect in bats suggest that acoustic alteration to vocal emissions may occur at the neural level (<2 s)^74,75^. With no other studies to refer to, a 2 s period was selected and the NL_BB calculated for this period immediately prior to each whistle. Although short, this period is longer than the average dolphin whistle recorded in the Fremantle Inner Harbour^76^.

GAMs were selected as they allow smoothing functions to be fitted to explanatory variables, generating curves which can flow more freely than a straight line for describing data. Although activity state and calf presence were included as factors and group size as a linear term, explanatory analyses indicated that NL_BB required fitting as a smoothed nonlinear term. A GAM with a logit-link binomial distribution was fitted for predicting the presence/absence of harmonics, whilst GAMs with a log-link Poisson distribution were fitted to predict counts of inflection points, extrema, breaks and saddles. The remaining whistle characteristics represented continuous data, and so were fitted with a log-link gamma distribution. These characteristics were whistle duration, minimum frequency, maximum frequency, delta frequency, start frequency and end frequency.

The appropriateness and assumptions for each model were assessed in R^71^. To verify independence of model errors, semi-variograms of standardised residuals were checked to ensure no temporal autocorrelation existed^77^. Variance inflation factors (VIFs^78^) were used to confirm the absence of concurvity (i.e. the non-parametric analogue of multi-collinearity) between explanatory variables. The absence of overdispersion was confirmed using the sum of squared Pearson residuals divided by sample size, minus the number of parameters.

Only the best fitting explanatory variables, chosen using the second-order Akaike information criterion corrected for small sample sizes (AICc^79^), were included in the final model for each whistle characteristic. Models with a difference of <2 AICc units are equally plausible as the best model to explain observed patterns in the data^79^. In such situations, the Akaike’s Information Criterion weight (wAICc) was calculated; the model with the greatest weight was then selected^79^. The QQ-plots, histograms, and percentage of deviance explained were examined to quantify each model’s goodness-of-fit^80^. To further investigate where differences existed between activity states for each whistle characteristic, post-hoc tests with Tukey contrasts were calculated from the fitted model to conduct pairwise comparisons.

All analyses of whistle characteristics were conducted in R^71^, using the packages: *car* ^81^; *MASS* ^82^; *mgcv* ^83^; *multcomp* ^84^; and *MuMIn* ^85^.

### Data Availability

The acoustic dataset analysed during the current study is available as a data descriptor under review in *Scientific Data* (SDATA-17-00084) doi:10.1038/sdata.2017.126. The visual datasets analysed during the current study are available from the corresponding author on reasonable request.

## Electronic supplementary material

Supplementary Information
