# Supplementary material for: Effects of vessel traffic and underwater noise on the movement, behaviour and vocalisations of bottlenose dolphins in an urbanised estuary
Source: Sci Rep. 2017 Oct 18;7:13437. doi: 10.1038/s41598-017-13252-z (PMC5647363; doi:10.1038/s41598-017-13252-z)

Effects of vessel traffic and underwater noise on the movement, behaviour and vocalisations of bottlenose dolphins in an urbanised estuary

Sarah A. Marley*, Chandra P. Salgado Kent, Christine Erbe and Iain M. Parnum

Centre for Marine Science and Technology (CMST), Curtin University, Perth, Western Australia

***Corresponding Author:** sarah.marley86@gmail.com

Supplementary Information

Supplementary Table S1 Definition of dolphin activity states (adapted from Shane et al. 1986 and Lusseau, 2003)

Activity State	Definition
Foraging (FO)	Dolphins involved in any effort to capture and consume prey, often involving quick, steep dives of long duration. Diving birds or jumping fish may also be observed.
Milling (MI)	Dolphins show frequent changes in heading, but stay in same location with no net movement.
Resting (RE)	Dolphins engaged in slow movements or ‘logging’ at the surface.
Socialising (SO)	Dolphins engaged in a diverse number of interactive behavioural events, including body contact, chasing, leaping, or hitting the water surface with body parts. Groups may split or join.
Travelling (TR)	Dolphins engaged in persistent, directional movement with short, relatively constant dive intervals.

Supplementary Table S2 Summary of the best fitting model for each whistle characteristic considering broadband noise levels, dolphin activity state, group size and calf presence. Explanatory variables with 'NA' were not included in the final model; ≤0.001 ***; ≤0.01 **; ≤0.05 *

**Duration**					
**Smooth Term**	**EDF**	**F**	**p-value**		
**NL_BB**	3.814	4.45	0.00107		**
**Parametric Terms**	**Parameter Estimate**	**Std. Error**	**t-value**	**p-value**	
**Intercept**	-1.27040	0.13895	-9.143	3.66e-16	***
**Activity State**					
Milling	-0.12007	0.11740	-1.023	0.3080	
Socialising	-0.74158	0.15895	-4.666	6.69e-06	***
Travelling	0.20874	0.08920	2.340	0.0206	*
**Group Size**	0.25221	0.05753	4.384	2.16e-05	***
**Calf Presence**	NA	NA	NA	NA	
**Min Frequency**					
**Smooth Term**	**EDF**	**F**	**p-value**		
**NL_BB**	3.26	6.187	0.000102		***
**Parametric Terms**	**Parameter Estimate**	**Std. Error**	**t-value**	**p-value**	
**Intercept**	8.30880	0.04726	175.793	< 2e-16	***
**Activity State**					
Milling	0.17376	0.07937	2.189	0.03010	*
Socialising	0.07199	0.09986	0.721	0.47205	
Travelling	0.17464	0.06088	2.869	0.00471	**
**Group Size**	NA	NA	NA	NA	
**Calf Presence**	-0.09619	0.05534	-1.738	0.08149	
**Max Frequency**					
**Smooth Term**	**EDF**	**F**	**p-value**		
**NL_BB**	3.14	21.46	1.08e-14		***
**Parametric Terms**	**Parameter Estimate**	**Std. Error**	**t-value**	**p-value**	
**Intercept**	9.37662	0.02303	407.127	< 2e-16	***
**Activity State**					
Milling	-0.14304	0.03867	-3.699	0.000302	***
Socialising	-0.13646	0.04846	-2.816	0.005506	**
Travelling	-0.11681	0.02961	-3.944	0.000122	***

**Group Size**	NA	NA	NA	NA	
**Calf Presence**	0.20377	0.02692	7.570	3.28e-12	***
**Delta Frequency**					
**Smooth Term**	**EDF**	**F**	**p-value**		
**NL_BB**	3.839	20.83	3.51e-16		***
**Parametric Terms**	**Parameter Estimate**	**Std. Error**	**t-value**	**p-value**	
**Intercept**	8.88953	0.05168	172.010	< 2e-16	***
**Activity State**					
Milling	-0.35244	0.08673	-4.064	7.72e-05	***
Socialising	-0.29466	0.11114	-2.651	0.00887	**
Travelling	-0.35491	0.06701	-5.297	4.06e-07	***
**Group Size**	NA	NA	NA	NA	
**Calf Presence**	0.45766	0.06094	7.510	4.7e-12	***
**Start Frequency**					
**Smooth Term**	**EDF**	**F**	**p-value**		
**NL_BB**	4.474	6.929	3.94e-06		***
**Parametric Terms**	**Parameter Estimate**	**Std. Error**	**t-value**	**p-value**	
**Intercept**	8.35023	0.04850	172.158	< 2e-16	***
**Activity State**					
Milling	0.15909	0.08120	1.959	0.05194	
Socialising	0.09588	0.10589	0.905	0.36667	
Travelling	0.18008	0.06320	2.849	0.00499	**
**Group Size**	NA	NA	NA	NA	
**Calf Presence**	-0.10563	0.05756	-1.835	0.06843	
**End Frequency**					
**Smooth Term**	**EDF**	**F**	**p-value**		
**NL_BB**	3.243	17.68	1.71e-12		***
**Parametric Terms**	**Parameter Estimate**	**Std. Error**	**t-value**	**p-value**	
**Intercept**	9.35074	0.03489	267.968	< 2e-16	***
**Activity State**					
Milling	-0.15346	0.05860	-2.619	0.00971	**
Socialising	-0.21389	0.07368	-2.903	0.00425	**
Travelling	-0.21882	0.04494	-4.869	2.77e-06	***
**Group Size**	NA	NA	NA	NA	

**Calf Presence**	0.23310	0.04084	5.707	5.80e-08	***
**Inflections**					
**Smooth Term**	**EDF**	**Chi-square**	**p-value**		
**NL_BB**	4.108	14.58	0.0139		*
**Parametric Terms**	**Parameter Estimate**	**Std. Error**	**t-value**	**p-value**	
**Intercept**	-0.29536	0.29108	-1.015	0.310256	
**Activity State**					
Milling	-0.76595	0.24421	-3.136	0.001710	**
Socialising	-1.23611	0.36062	-3.428	0.000609	***
Travelling	0.04023	0.15075	0.267	0.789573	
**Group Size**	0.45154	0.11981	3.769	0.000164	***
**Calf Presence**	0.61350	0.13797	4.446	8.73e-06	***
**Extrema**					
**Smooth Term**	**EDF**	**Chi-square**	**p-value**		
**NL_BB**	3.353	26.12	3.84e-05		***
**Parametric Terms**	**Parameter Estimate**	**Std. Error**	**t-value**	**p-value**	
**Intercept**	-2.6727	0.4758	-5.618	1.94e-08	***
**Activity State**					
Milling	0.8539	0.613	1.851	0.064129	
Socialising	-1.3227	0.7810	-1.694	0.090323	
Travelling	1.6834	0.3735	4.508	6.56e-06	***
**Group Size**	0.5887	0.1655	3.558	0.000374	***
**Calf Presence**	NA	NA	NA	NA	
**Harmonics**					
**Smooth Term**	**EDF**	**Chi-square**	**p-value**		
**NL_BB**	7.306	16.04	0.0455		*
**Parametric Terms**	**Parameter Estimate**	**Std. Error**	**t-value**	**p-value**	
**Intercept**	-2.7016	0.6562	-4.117	3.83e-05	***
**Activity State**					
Milling	3.0940	0.8174	3.785	0.000154	***
Socialising	0.8052	1.0343	0.778	0.436307	
Travelling	2.5907	0.7374	3.513	0.000442	***
**Group Size**	NA	NA	NA	NA	
**Calf Presence**	NA	NA	NA	NA	

Supplementary Table S3 Summary of the best fitting model for each whistle characteristic, considering octave-band levels (OBLs) centred around 1 kHz (NL_1000), 16 kHz (NL_16000), and 32 kHz (NL_32000). Explanatory variables with 'NA' were not included in the final model; ≤0.001 ***; ≤0.01 **; ≤0.05 *

**Duration**					
**Parametric Terms**	**Parameter Estimate**	**Std. Error**	**t-value**	**p-value**	
**Intercept**	-0.65978	0.03453	-19.11	< 2e-16	***
**Smooth Terms**	**EDF**	**F**	**p-value**		
**NL_1000**	8.423	7.810	9.84e-10		***
**NL_16000**	1.000	3.820	0.052602		
**NL_32000**	8.000	3.532	0.000665		***

**Min Frequency**					
**Parametric Terms**	**Parameter Estimate**	**Std. Error**	**t-value**	**p-value**	
**Intercept**	8.3862	0.0213	393.8	< 2e-16	***
**Smooth Terms**	**EDF**	**F**	**p-value**		
**NL_1000**	8.103	10.024	5.02e-12		***
**NL_16000**	4.765	1.675	0.114		
**NL_32000**	NA	NA	NA		

**Max Frequency**					
**Parametric Terms**	**Parameter Estimate**	**Std. Error**	**t-value**	**p-value**	
**Intercept**	9.37007	0.01236	758.0	< 2e-16	***
**Smooth Terms**	**EDF**	**F**	**p-value**		
**NL_1000**	3.808	25.40	< 2e-16		***
**NL_16000**	1.000	20.17	1.34e-05		***
**NL_32000**	NA	NA	NA		

**Delta Frequency**					
**Parametric Terms**	**Parameter Estimate**	**Std. Error**	**t-value**	**p-value**	
**Intercept**	8.84362	0.02947	300.1	< 2e-16	***
**Smooth Terms**	**EDF**	**F**	**p-value**		
**NL_1000**	3.868	19.24	5.20e-05		***
**NL_16000**	NA	NA	NA		
**NL_32000**	1.000	17.28	5.23e-05		***

**Start Frequency**					

Parametric Terms	Parameter Estimate	Std. Error	t-value	p-value	
Intercept	8.4294	0.0228	369.7	< 2e-16	***
Smooth Terms	EDF	F	p-value		
NL_1000	8.092	10.11	3.62e-12		***
NL_16000	3.528	1.58	0.188		
NL_32000	NA	NA	NA		
**End Frequency**					
Parametric Terms	Parameter Estimate	Std. Error	t-value	p-value	
Intercept	9.29580	0.01806	514.6	< 2e-16	***
Smooth Terms	EDF	F	p-value		
NL_1000	5.045	14.148	1.29e-13		***
NL_16000	NA	NA	NA		
NL_32000	1.428	9.049	0.000271		***
**Inflections**					
Parametric Terms	Parameter Estimate	Std. Error	t-value	p-value	
Intercept	0.89326	0.05163	17.3	< 2e-16	***
Smooth Terms	EDF	Chi-square	p-value		
NL_1000	7.902	19.253	0.0169		*
NL_16000	2.824	9.063	0.0599		
NL_32000	8.483	18.271	0.0267		*
**Extrema**					
Parametric Terms	Parameter Estimate	Std. Error	t-value	p-value	
Intercept	-0.3372	0.1702	-1.981	0.0476	*
Smooth Terms	EDF	Chi-square	p-value		
NL_1000	6.753	49.354	2.49e-08		***
NL_16000	2.108	3.846	0.19614		
NL_32000	1.000	10.734	0.00105		**
**Harmonics**					
Parametric Terms	Parameter Estimate	Std. Error	t-value	p-value	
Intercept	-1.5032	0.7219	-2.082	0.0373	*
Smooth Terms	EDF	Chi-square	p-value		

**NL_1000**	6.847	21.969	0.01558	*
**NL_16000**	1.000	5.974	0.01452	*
**NL_32000**	4.089	16.820	0.00514	**

Supplementary Figure S4 Results of the nine whistle GAMs which selected the 1 kHz octave-band level (NL_1000) as a significant explanatory variable.

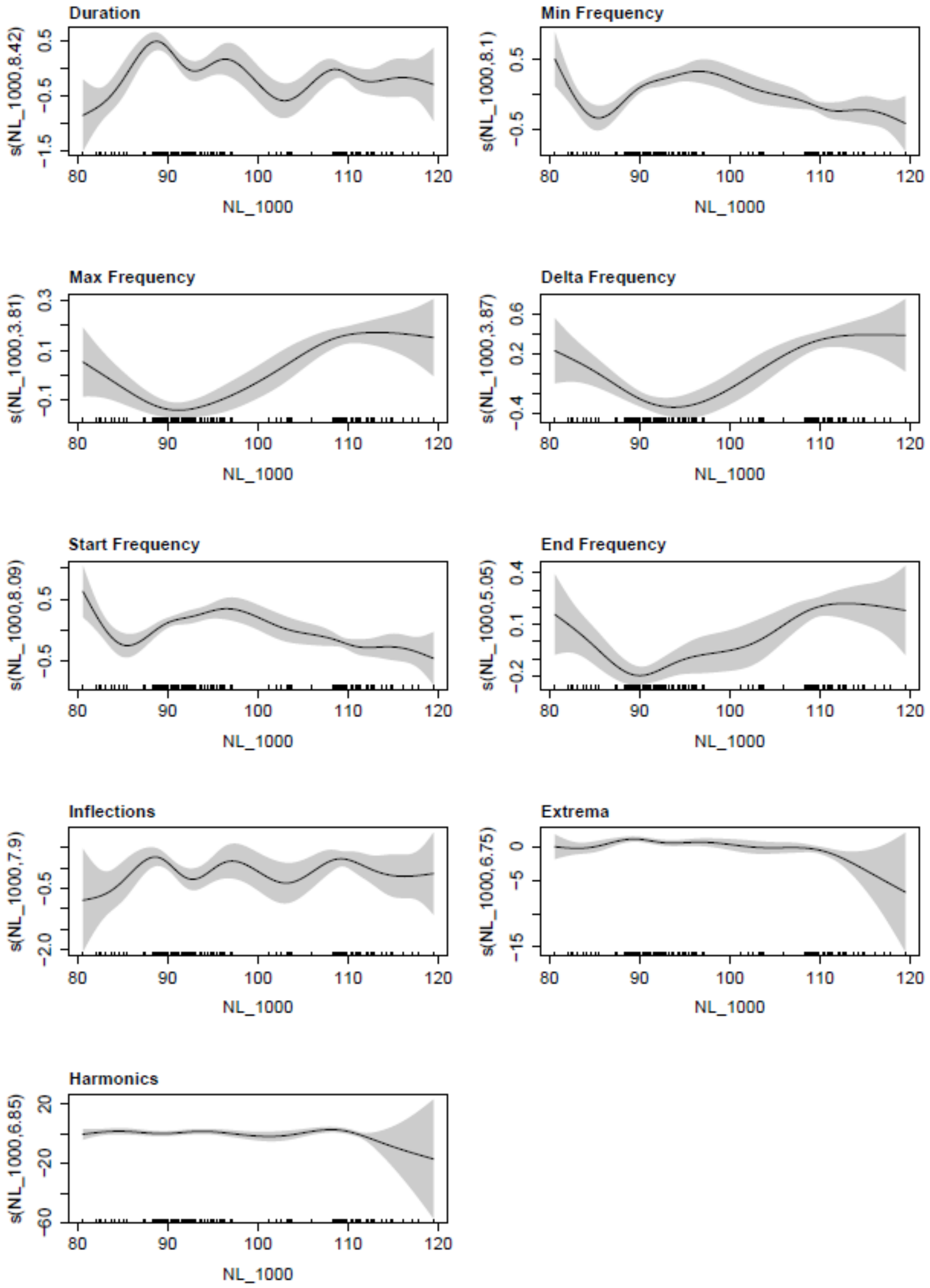

Supplementary Figure S5 Results of the three whistle GAMs which selected the 16 kHz octave-band level (NL_16000) as a significant explanatory variable.

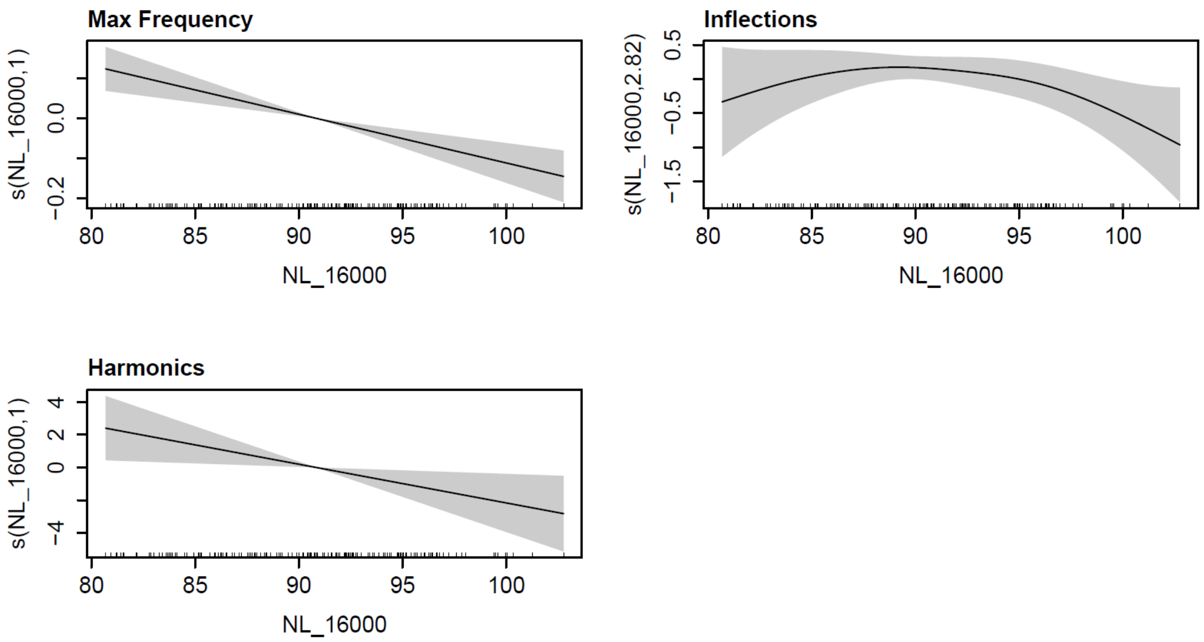

Supplementary Figure S6 Results of the six whistle GAMs which selected the 32 kHz octave-band level (NL_32000) as a significant explanatory variable.

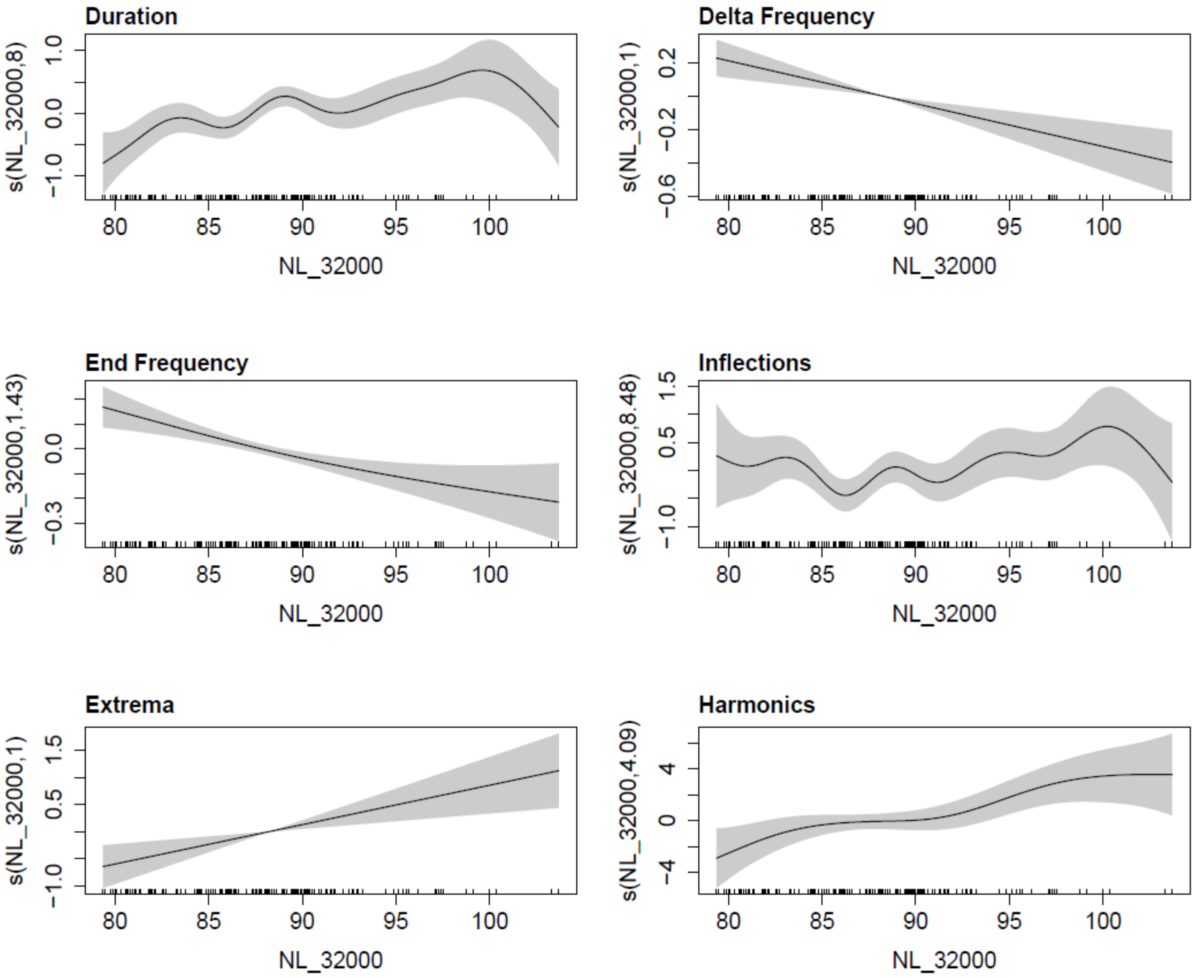

Supplement: Supplementary file 1 — Supplementary Information [file 41598_2017_13252_MOESM1_ESM.pdf]
